# EXPLORING THE PHYSICAL FUNCTION PROFILES OF OLDER ADULTS WITH SERIOUS MENTAL HEALTH CONDITIONS

**DOI:** 10.1093/geroni/igac059.1893

**Published:** 2022-12-20

**Authors:** Julia Browne, Miriam Morey, Eric Elbogen, Kim Mueser, Katherine Hall

**Affiliations:** Durham VA Health Care System, Durham, North Carolina, United States; Durham VA Health Care System, Durham, North Carolina, United States; Durham VA Health Care System, Durham, North Carolina, United States; Boston University, Boston, Massachusetts, United States; Durham VA Health Care System, Durham, North Carolina, United States

## Abstract

Older adults with serious mental health conditions experience high rates of comorbid chronic medical diseases, which affect the physical function and long-term aging trajectory of this population. Existing physical function research in this population has focused almost exclusively on endurance with little to no attention paid to other important domains such as balance, mobility, and strength, which are critical to promoting independence and improved quality of life. The primary aim of this study was to explore the multiple dimensions of function that are important to maintaining independence and health in older adults with serious mental health conditions. This study examined publicly available data from the 2014 and 2016 waves of the Health and Retirement Study, a national longitudinal panel study of older adults. Analyses were limited to participants that self-reported lifetime diagnoses of serious mental health conditions (schizophrenia, bipolar disorder, depression, posttraumatic stress disorder). The sample comprised 428 older adults over age 50 (M=67.1, SD=10.1; 68% female) who completed standardized assessments of grip strength, single-leg standing balance, and usual walking speed. Scores on these three measures were compared to established age- and/or gender-based norms for each assessment to characterize performance. Overall, 65% of the sample demonstrated deficits in strength and 31% of the sample performed below established norms for both balance and walking speed. These findings highlight that physical function in this population is compromised across multiple domains and suggest a need for multicomponent interventions.

